# Rational Phosphorus Application Facilitates the Sustainability of the Wheat/Maize/Soybean Relay Strip Intercropping System

**DOI:** 10.1371/journal.pone.0141725

**Published:** 2015-11-05

**Authors:** Yuanxue Chen, Tao Zhou, Chaochun Zhang, Ke Wang, Jing Liu, Junyu Lu, Kaiwei Xu

**Affiliations:** 1 Department of Plant Nutrition and Microbiology, College of Resource Science, Sichuan Agricultural University, Chengdu, China; 2 Department of Plant Nutrition, College of Resource and Environmental Sciences, China Agricultural University, Beijing, China; 3 Ya’an Agricultural Bureau, Ya’an, China; Chinese Academy of Sciences, CHINA

## Abstract

Wheat (*Triticum aestivum* L.)/maize (*Zea mays* L.)/soybean (*Glycine max* L.) relay strip intercropping (W/M/S) system is commonly used by the smallholders in the Southwest of China. However, little known is how to manage phosphorus (P) to enhance P use efficiency of the W/M/S system and to mitigate P leaching that is a major source of pollution. Field experiments were carried out in 2011, 2012, and 2013 to test the impact of five P application rates on yield and P use efficiency of the W/M/S system. The study measured grain yield, shoot P uptake, apparent P recovery efficiency (PRE) and soil P content. A linear-plateau model was used to determine the critical P rate that maximizes gains in the indexes of system productivity. The results show that increase in P application rates aggrandized shoot P uptake and crops yields at threshold rates of 70 and 71.5 kg P ha^-1^ respectively. With P application rates increasing, the W/M/S system decreased the PRE from 35.9% to 12.3% averaged over the three years. A rational P application rate, 72 kg P ha^-1^, or an appropriate soil Olsen-P level, 19.1 mg kg^-1^, drives the W/M/S system to maximize total grain yield while minimizing P surplus, as a result of the PRE up to 28.0%. We conclude that rational P application is an important approach for relay intercropping to produce high yield while mitigating P pollution and the rational P application-based integrated P fertilizer management is vital for sustainable intensification of agriculture in the Southwest of China.

## Introduction

Food security, resource saving and environmental safety are focuses of globally many governments’ attention, especially Chinese government. During the period 1960 to 2008, the total grain production of China increased 3.4 fold from 110 to 483 million tons [1], but P fertilizer input increased 91 fold during the same period [2]. Grain production greatly increased because of this substantial P fertilizer input, but excessive P use by farmers led to low P use efficiency, high environmental risk and P accumulation in soils which occurred in most regions of China [3–4]. In the North China Plain, a major area for intensive crop production, the annual net P input was 53 kg ha^-1^ [5]. In China, the total net P input during the period from 1980 to 2007 was 242 kg ha^-1^, causing soil Olsen-P increase from 7.4 to 24.7 mg kg^-1^ [6] while the P recovery efficiency (PRE) is currently only 15–20% [7–8]. Zhong [9] considered 20 mg kg^-1^ to be the Olsen-P threshold for optimal plant growth and 40 mg kg^-1^ to be the critical level for having risk of P leaching in many Chinese soil types. However, about 9.3% of China’s arable land exceeded 40 mg kg^-1^ of Olsen-P in soil [6] and about 60% of inland lakes showed eutrophication, and 67% of the P source resulting in water pollution was derived from agriculture [10].

Many studies report that interspecific facilitation results in overyielding of intercropping relative to monocultures, and also increase resource (solar, water and nutrients) use efficiency [11–13]. In the Southwest China, one of the most densely populated agricultural regions, annual planting systems include rotation and intercropping [14], and the dominant cropping system is wheat/maize/soybean (W/M/S) relay strip intercropping. In this system, wheat and soybean are rotated as a double crop in the same strip, where wheat is planted and harvested first, and then soybean is planted. The fertilizer residue remained in the soil after wheat harvest can be utilized by the subsequent soybean. The wheat-soybean double crop strip is relay intercropped with maize grown in a different strip. Interspecific interactions, such as facilitation and competition, occur primarily between the different strips, so between wheat and maize, and then between soybean and maize. Most studies on the W/M/S system examined the impacts of different variety combinations [15] and nitrogen (N) application [16–17] on productivity. However, as yet no work has focused on P fertilizer management considering both soil P status and cropping system’s annual productivity. Rational P management to reduce P leaching is vital in the Southwest of China as this area locates the upstream of the Yangtze River and the arable field on the hilly landscape is easily eroded by rainfall [14].

The scientific problem of the current study is what P application rate is rational to produce high grain yield of wheat, maize, soybean and the W/M/S system, and to achieve high PRE and stable soil P level in the Southwest of China. A three-year field experiment was conducted to test the hypothesis that a rational P application in the W/M/S system maximizes productivity, enhances PRE, and generates a net balance between P input and output.

## Materials and Methods

### Site description

**Fig 1:**
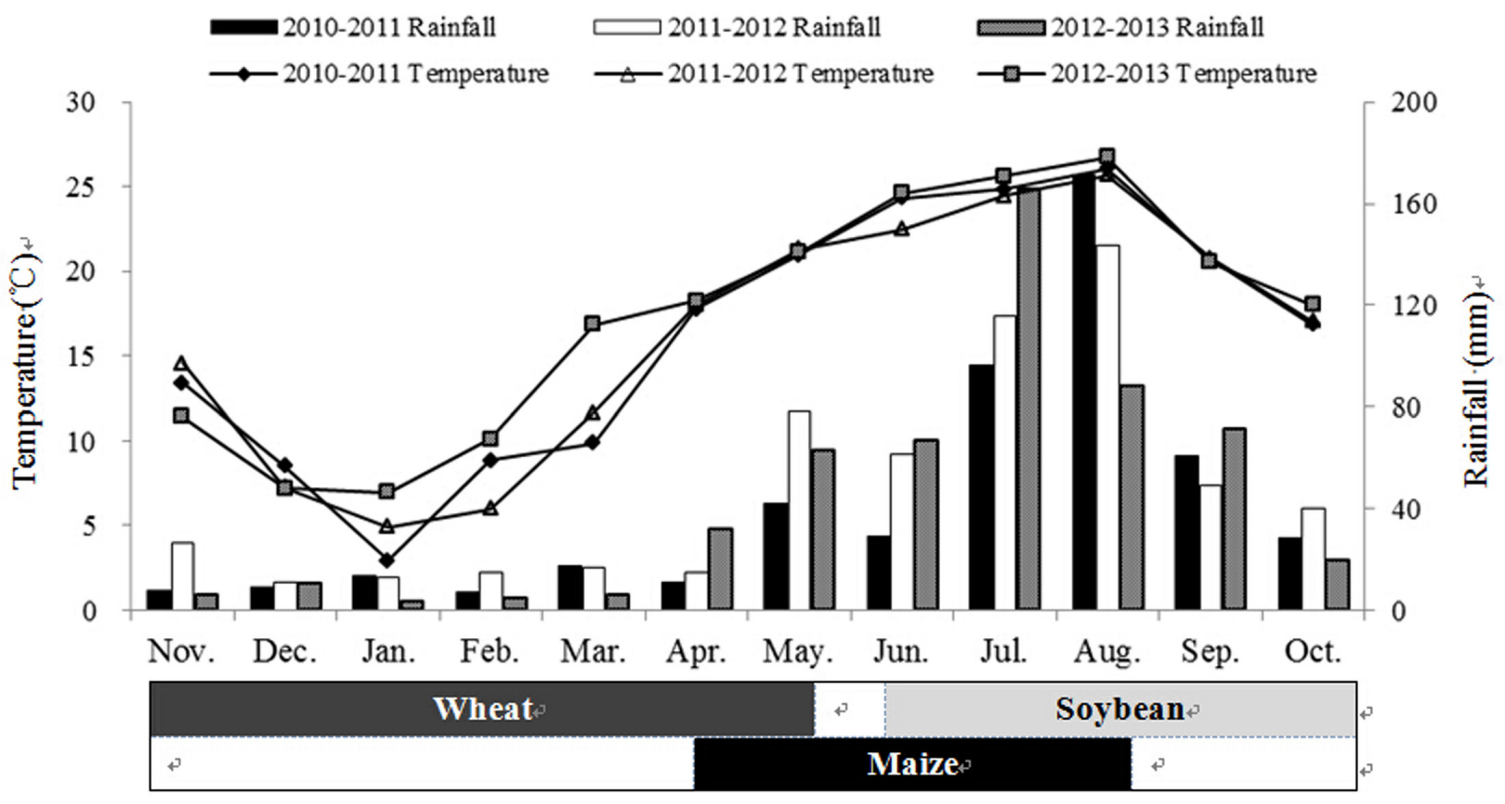
Monthly average rainfall (mm) and temperature (℃) at the experimental spot in 2011, 2012 and 2013 and planting/harvest times of the three crops during growth period. (1) Shading indicates that the crops are growing in the field; (2) Wheat intercropped with maize has a co-growth period of almost 45 days; (3) Maize intercropped with soybean has a co-growth period of almost 55 days.

The field experiments were conducted in 2011, 2012 and 2013 at the Ya’an Experimental Station (29°58′N, 102°58′E) with an altitude of 600 m above sea level, Sichuan Province, China. Annual mean temperature is 15.4°C with a maximum and minimum temperature of 25.4°C and 6.1°C respectively. The frost-free period is 294 days, annual precipitation is 1500 mm and potential evaporation is 838 mm. Annual sunshine is 1019 hours and total solar radiation averages 3,750 MJ m^-2^ yr^-1^. The monthly average temperature and rainfall values during the current experimental period showed that generally the temperature and rainfall are the lowest in January and highest in August (Fig 1). The experimental soil is classified as a Purple soil (Luvic Xerosols, FAO classification). At the start of the study the soil pH (water) was 6.2, organic matter content 32.1 g kg^-1^, total N 2.10 g kg^-1^, available N 112 mg kg^-1^, Olsen-P 13.2 mg kg^-1^, exchangeable K 71 mg kg^-1^, and Cation Exchange Capacity 21.5 cmol kg^-1^ of dry soil in the top 20 cm soil layer. The proportion of soil particles and bulk density [18] in the soil profile are presented in Table 1.

**Table 1:** Particle size distribution and bulk density of the experimental soil change with soil depth.

Soil layer (cm)	Proportions (%) of soil particles				Texture (FAO classification)	Bulk density (g cm^-3^)
	>2.0 mm	2.0–0.02 mm	0.02–0.002 mm	<0.002 mm		
**0–20**	0	56.0	26.0	18.0	Sandy clay loam	1.48
**20–40**	0	52.0	24.0	24.0	Clay loam	1.53
**40–60**	0	46.0	30.0	24.0	Clay loam	1.39
**60–80**	0	46.0	28.0	26.0	Loamy clay	1.39
**80–100**	0	46.0	28.0	26.0	Loamy clay	1.39

### Experiment design and crop management

**Fig 2:**
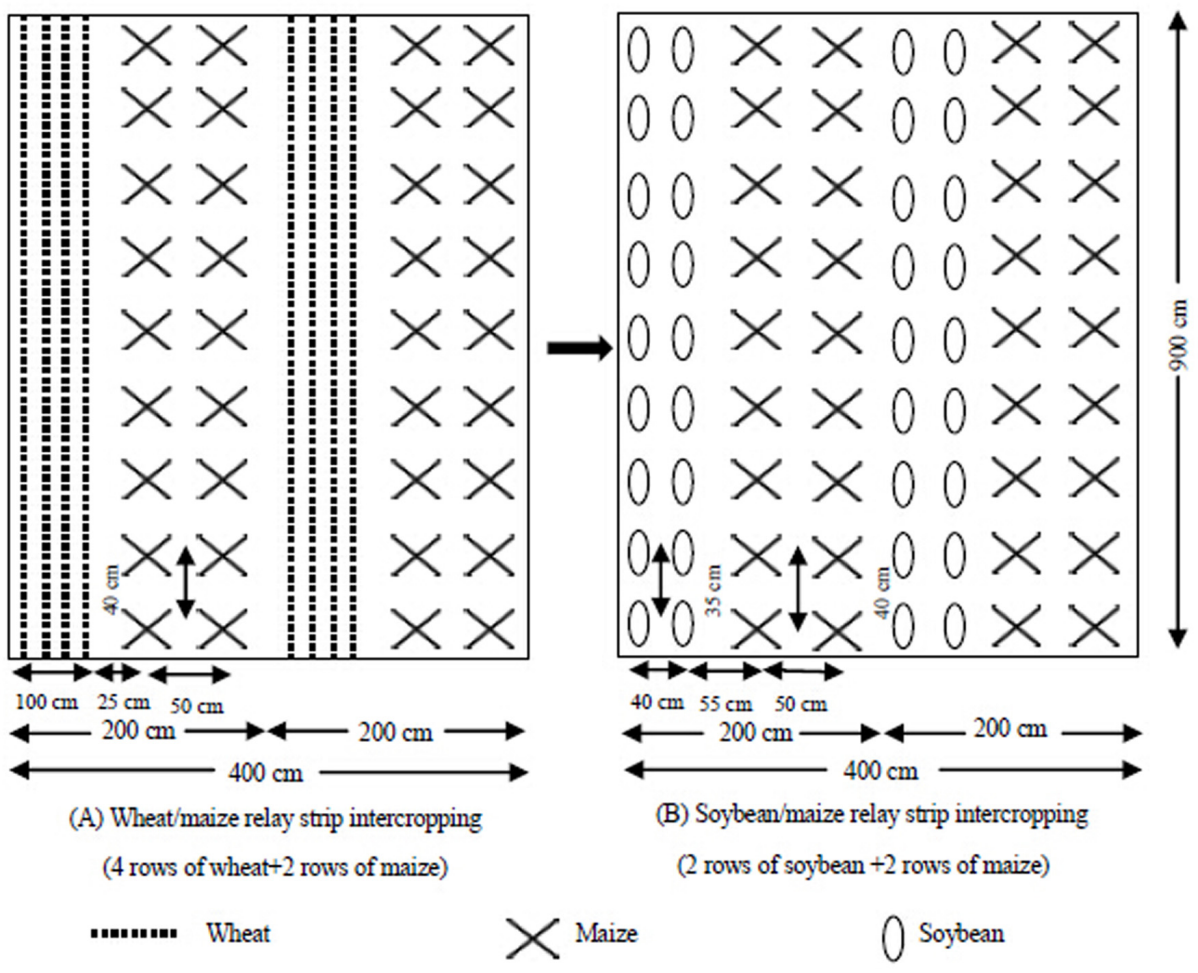
Diagram showing the arrangement of wheat intercropped with maize (A) and maize intercropped with soybean (B) in the field plot.

The field experiment was designed as a randomized block with five P treatment levels and four replicates (blocks) over three years in the same location. Every block consisted of five plots measuring 4 × 9 m^2^ each in which wheat (*Triticum aestivum* L. cv. Chuanmai No. 37), maize (*Zea mays* L. cv. Chuandan No. 418) and soybean (*Glycine max* L. cv. Gongxuan No.1) were cultivated to make the wheat/maize/soybean (W/M/S) strip relay intercropping system (S1 Fig). Each plot included two duplicates of one planting unit which was consisted of a 1-m wide wheat-soybean strip and an identical width maize strip. Each double crops strip had four rows of wheat followed by two rows of soybean after wheat was harvested, and each maize strip included two rows of maize (Fig 2). Wheat was sown in rows at spacing of 25 cm between rows. Two maize seedlings were transplanted per hole at a spacing of 40 cm between holes and 50 cm between rows. Soybean was directly sown after wheat harvested without soil tillage at spacing of 35 cm between plants and 40 cm between rows. The space was 25 cm between wheat and maize and 55 cm between maize and soybean (Fig 2). Density of intercropped wheat was about 240 plants m^-2^, maize 5 plants m^-2^, soybean 5.7 plants m^-2^. Wheat was sown on 11^th^ November in 2010, 11^th^ November in 2011, and 10^th^ November in 2012 and harvested on 25^th^ May in 2011, 30^th^ May in 2012, and 11^th^ May in 2013 respectively. Maize was transplanted on 16^th^ April in 2011, 15^th^ April in 2012, and 7^th^ April in 2013 and harvested on 10^th^ August in 2011, 6^th^ August in 2012, and 6^th^ August in 2013 respectively. Soybean was sown on 15^th^ June in 2011, 14^th^ June in 2012, and 12^th^ June in 2013 and harvested on 27^th^ October in 2011, 30^th^ October in 2012, and 5^th^ November in 2013 respectively. The coexisting periods of wheat with maize, and maize with soybean were approximately 45 days and 55 days respectively (Fig 1). With regard to soil tillage, before the experiment of annually W/M/S system i.e. before wheat sowing or after soybean harvested every year, the soil of whole experiment including both wheat-soybean strip and maize strip were identically turned over to 20 cm in depth and then smashed and plattened all by hand. During the experiment of annually W/M/S system there is no soil tillage.

The P rates marked as P_0_, P_1_, P_2_, P_3_, P_4_, for wheat were 0, 20, 40, 60 and 80 kg P ha^-1^ and the rates for maize were 0, 16, 32, 48 and 64 kg P ha^-1^ applied as triple superphosphate. The total N application rate for wheat and maize were 120 kg N ha^-1^ and 225 kg N ha^-1^ as urea respectively. The K application rate for wheat and maize were 75 kg K ha^-1^ and 87 kg K ha^-1^ respectively as potassium chloride. Soybean as the crop following wheat had no fertilizer input while utilizing the residual nutrients of fertilizers left by wheat (Table 2). All of the P and K fertilizers were applied as basal fertilizer before wheat or maize sowing, but N fertilizer was split to a basal fertilization and two topdressings which were applied to wheat at the tillering stage and the stem elongation stage, and applied to maize at the stem elongation stage and the tasseling stage. The proportions of basal fertilizer and two topdressings were 40-30-30 in the percentage of the total N fertilizer regarding wheat and 30-30-40 regarding maize. No organic manure was applied. During the growth period all the plots were well irrigated and weeded manually or by chemical control.

**Table 2:** P application rates for wheat, maize and soybean in the W/M/S system (kg P ha^-1^ year^-1^).

Treatment	Wheat	Maize	Soybean	Wheat-Soybean strip	W/M/S
**P**_**0**_	0	0	0	0	0
**P**_**1**_	20	16	0	20	36
**P**_**2**_	40	32	0	40	72
**P**_**3**_	60	48	0	60	108
**P**_**4**_	80	64	0	80	144

### Plant sampling and analysis

Shoot biomass, including stem, leaves, and grain, of wheat, maize, and soybean were measured at maturity. Five plants of maize, 10 plants of soybean and 0.4 ×1.0 m^2^ of wheat were sampled from the middle strip of each plot. The samples were oven dried at 70°C and then ground for further chemical measurements. The samples were wet-digested with concentrated H_2_SO_4_ and H_2_O_2_ (30%), and then N content was measured with the micro-Kjeldahl procedure, and P determined by the vanadomolybdate method [19]. Shoot P uptake was calculated by multiplying P concentration with the shoot biomass. Grain yield of the three crops came from harvesting the remaining parts of the plot after shoot sampling.

### Soil sampling and analysis

In 2010 before crops were planted, soil samples were taken with a soil cores to a depth of 1 m and then sliced to 0–20 cm, 20–40 cm, 40–60 cm, 60–80 cm and 80–100 cm layers. The soil samples from all layers were analyzed for particle size distribution and bulk density. The 0–20 cm soil subsamples were analyzed for their physiochemical properties, including pH, organic matter, total N, available N, Olsen-P, exchangeable K and cation exchange capacity [20]. At harvest of maize and soybean, soil samples from each plot were collected from the 0–20 cm layer in 2011 and 2012, but in 2013 at harvest of maize and soybean one-meter deep soil cores were taken from each plot and similarly sliced to 0–20 cm, 20–40 cm, 40–60 cm, 60–80 cm, and 80–100 cm layers. Two subsamples per layer from each plot were taken and mixed uniformly to give one pooled sample. Soil samples were air-dried and passed through a 2.0 mm mesh sieve. A 2.5 g soil sample was shaken with 50 ml of 0.5 mol L^-1^ NaHCO_3_ for 30 minutes at 25°C, and after the suspension was filtered, the P concentration of the filtrate was determined using the molybdate ascorbic acid method [20]

### Calculations

The PRE is defined as percentage recovery of fertilizer P [21] and has been generally accepted as an agronomically sound index to evaluate the P absorption efficiency of crops from P fertilizer applied to soil. PRE is calculated according to:

$$\text{PRE}\;\left(\text{\%}\right)=\frac{U_{f}-U_{0}}{P_{f}}\times 100$$ (1)

where *U*_*f*_ is P uptake of the P treated crops, *U*_*0*_ is P uptake of the no-P treated crops, and *P*_*f*_ is the amount of P fertilizer applied. Considering the experiment was continuously carried on for 3 years in the same location, we calculated the PRE for wheat (PRE_w_), maize (PRE_m_), wheat-soybean strip (PRE_w-s_), and W/M/S system (PRE_w/m/s_) by the same principle. For example PRE_w_ was calculated as:PRE_W_ = 100×(*U*_*w−f*_−*U*_*w−0*_)/*P*_*w−f*_, where *U*_*w−f*_ is the mean of P uptake of the P treated wheat in 2011, 2012 and 2013; *U*_*w−0*_ is the mean of P uptake of the none-P treated wheat in 2011, 2012 and 2013 *P*_*w−f*_ is the mean of P fertilizer rates applied to wheat in 2011, 2012 and 2013.

Apparent P balance (APB) was calculated from the magnitude of applied P minus the total P uptake by the aboveground biomass of wheat (APB_w_), maize (APB_m_), wheat-soybean strip (APB_w-s_) and W/M/S system (APB_w/m/s_) respectively [22].

The linear-plateau model was used to analyze the relationship between P application rate and yield, aboveground biomass and shoot P uptake. The linear-plateau model is defined by Eqs (2) and (3) as:

 y=a+bx     if(x)<c (2)

 y=Yp            if(x)≥c (3)

where *y* is yield, aboveground biomass or shoot P content; *a* is the intercept parameter; *b* is the slope parameter; *x* is the P application rate (kg P ha^-1^); *c* is the critical P application rate (kg P ha^-1^), which is the interception point of the two linear segments; and *Yp* is the plateau value which is often 90% of the maximum yield [23]. Eq 2 can be interpreted as the region during which the crop responds to P application, and Eq 3 to the plateau region where additional P application has no effect.

### Statistical analysis

Regression equations were developed for the relationships between P application rates and yield, aboveground biomass, or shoot P uptake for the linear-plateau model using the SAS 9.1.3 software (SAS Institute Inc., USA). Significance differences in PRE and soil Olsen-P content between the different P treatments were conducted at *p*<0.05 level by least significant difference (LSD) using the SPSS 19.0 software (SPSS Institute Inc., USA).

## Results

### Shoot biomass and grain yield of the W/M/S system

**Fig 3:**
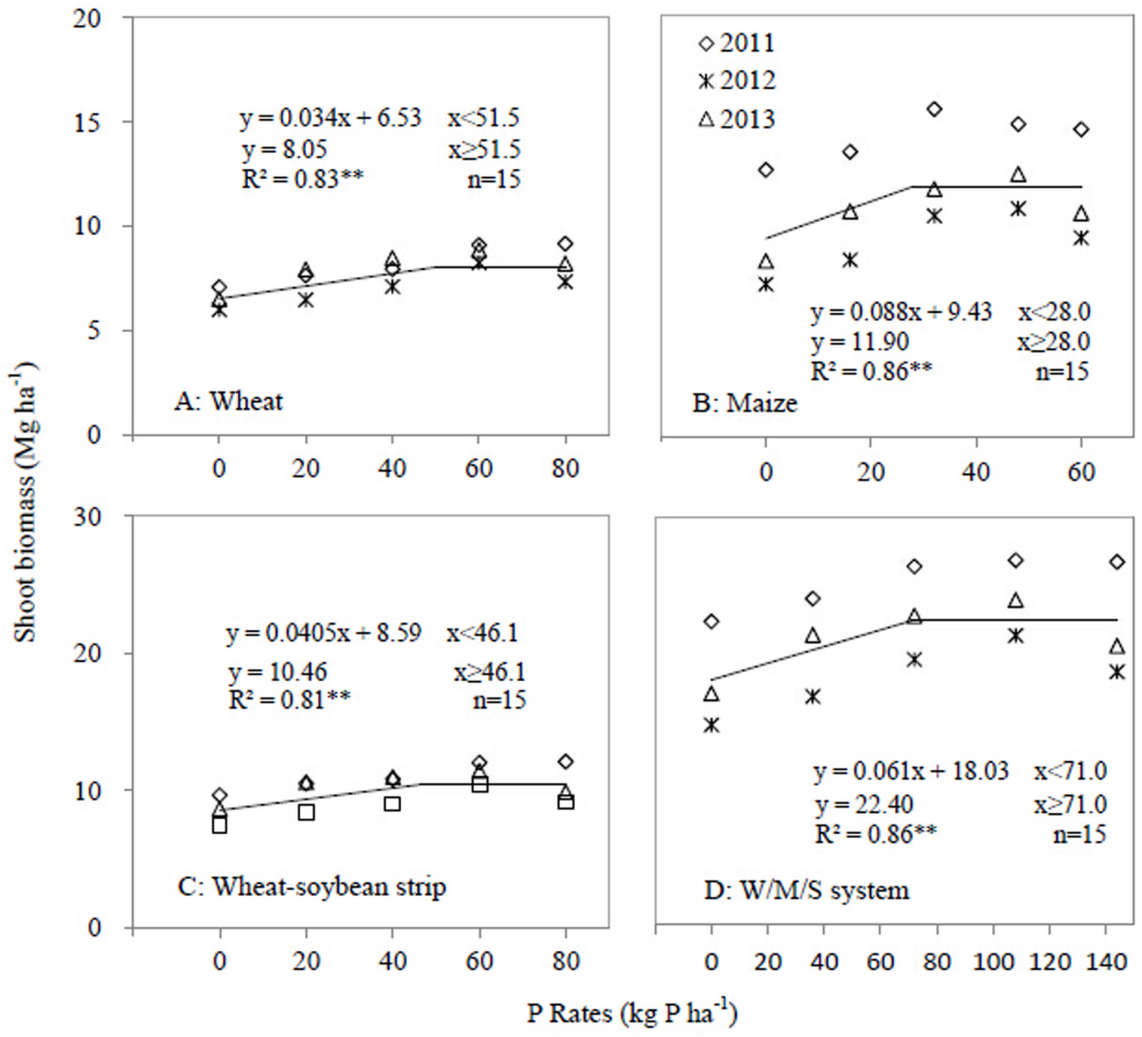
Shoot biomass as affected by P application rates in 2011, 2012 and 2013. A, Wheat; B, Maize; C, Wheat-soybean strip; D, W/M/S system. Each data point was the mean of four replicates.

**Fig 4:**
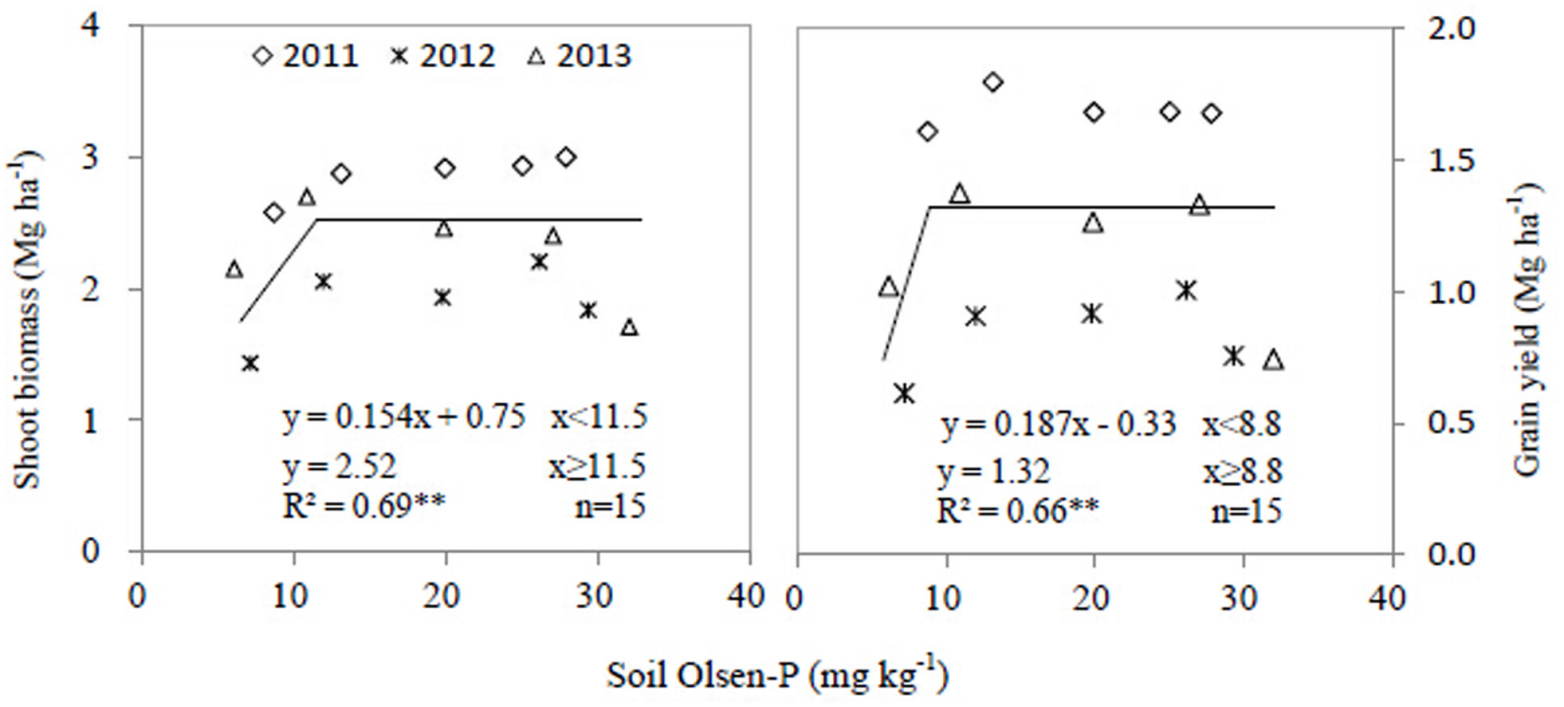
Shoot biomass and grain yield of soybean response to soil Olsen-P content. Each data point was the mean of four replicates.

The linear-plateau model denoted linear relationships between P application rates and shoot biomass of wheat (R^2^ = 0.83), maize (R^2^ = 0.86), wheat-soybean strip (R^2^ = 0.81), and W/M/S system (R^2^ = 0.86) (*P*<0.001) (Fig 3). Increase in P application rate increased shoot biomass, but the critical application rates for crop shoot biomass were greatly affected by crop species and P application rate (Fig 3). For wheat, maize and wheat-soybean strip, the critical P application rate was 51.5, 28.0 and 46.1 kg P ha^-1^ respectively, and the plateau shoot biomass was 8.05, 11.90 and 10.46 Mg ha^-1^ respectively. The critical P application rate for wheat-soybean strip was lower than that for wheat. The critical P rate for the W/M/S system was 71.0 kg P ha^-1^, which is 3.1 kg P ha^-1^ lower than the summed critical rate of both wheat-soybean and maize strips (Fig 3). Regarding soybean shoot biomass, the critical soil Olsen-P value was 11.5 mg kg^-1^ and the plateau value was 2.52 Mg ha^-1^ (Fig 4).

**Fig 5:**
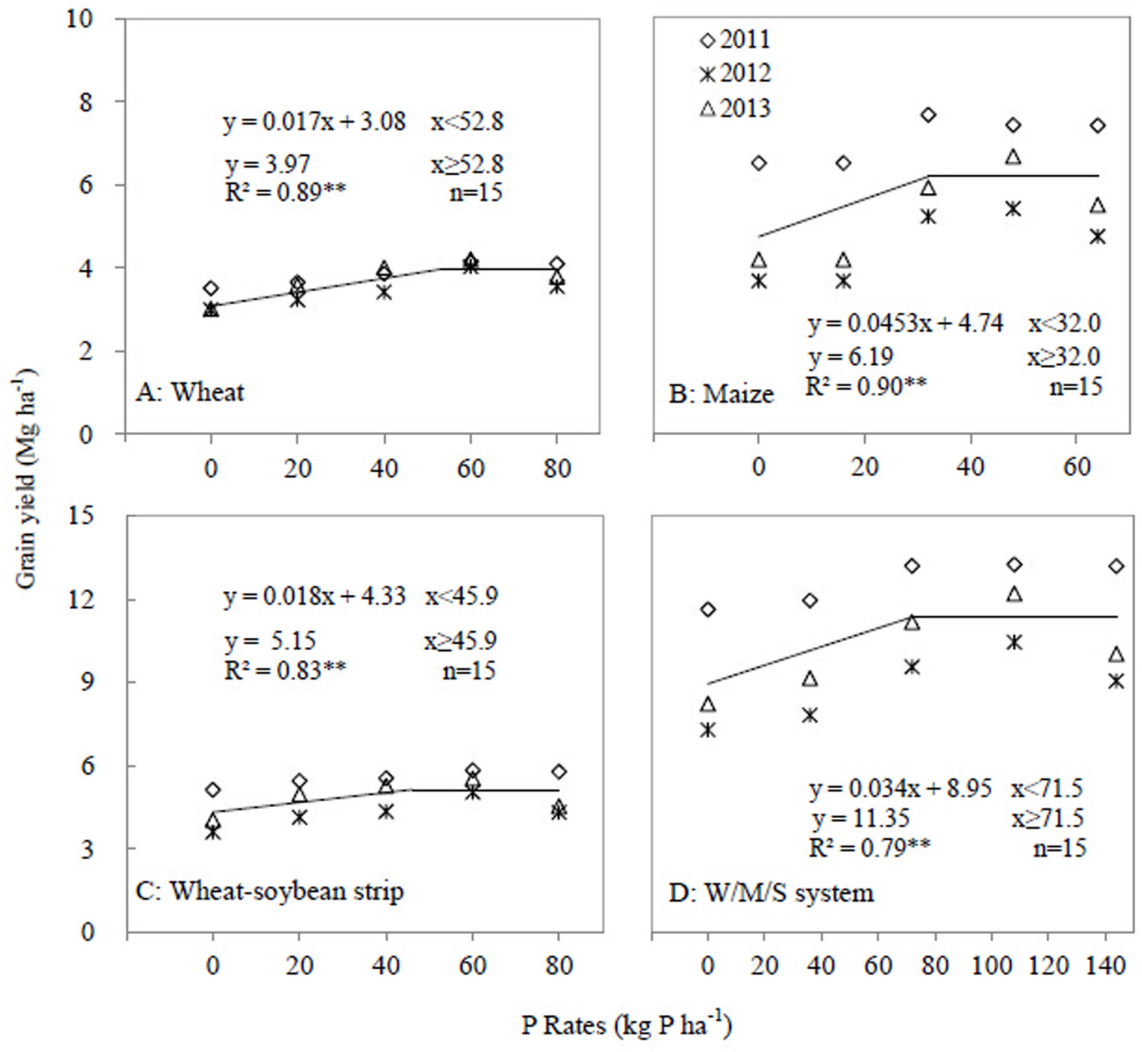
Grain yield as affected by P application rates in 2011, 2012 and 2013. A, Wheat; B, Maize; C, Wheat-soybean strip; D, W/M/S system. Each data point was the mean of four replicates.

Similarly, the linear-plateau model presented the linear relationships between the P application rate and yield of wheat (R^2^ = 0.89), maize (R^2^ = 0.90), wheat-soybean strip (R^2^ = 0.83) and the W/M/S system (R^2^ = 0.79) (*P*<0.001) (Fig 5). For grain yield, a critical P application rate was shown for wheat, maize, wheat-soybean strip and W/M/S system (Fig 5). Increase in P application rate increased grain yield, but the critical P application rates were affected by crop species and cropping systems. For wheat, maize and wheat-soybean strips, the critical P rates were 52.8, 32.0 and 45.9 kg P ha^-1^ respectively, and the plateau grain yield was 3.97, 6.19 and 5.15 Mg ha^-1^ respectively (Fig 5). The critical P rate for the W/M/S system was 71.5 kg P ha^-1^, which is 6.4 kg P ha^-1^ lower than the summed critical rate of both wheat-soybean and maize strip. The plateau grain yield of the W/M/S system was 11.35 Mg ha^-1^ (Fig 5). Considering the soybean grain yield, the critical soil Olsen-P value was 8.8 mg kg^-1^ and the plateau value was 1.32 Mg ha^-1^ (Fig 4).

### Shoot P uptake by intercropped species

**Fig 6:**
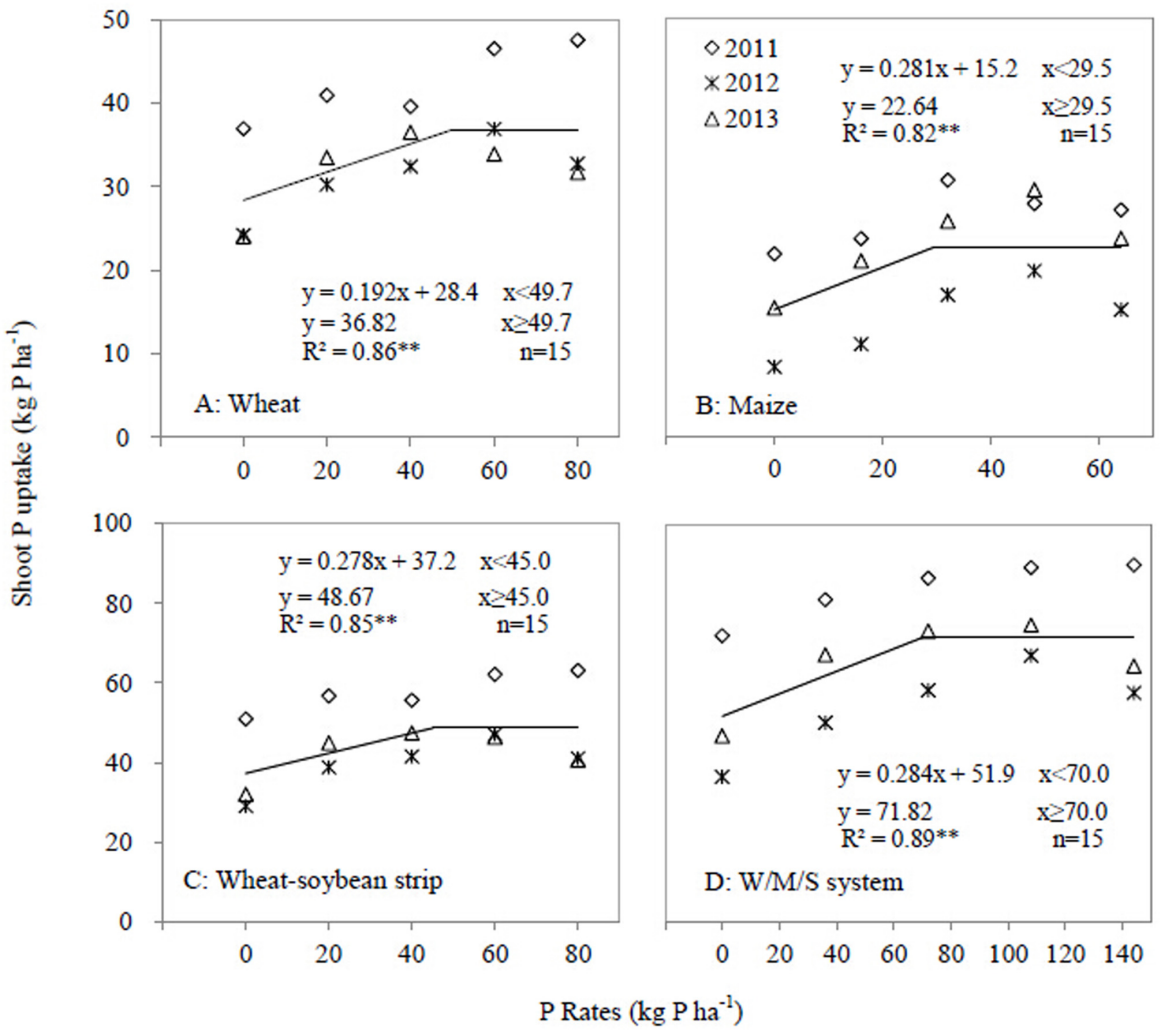
Shoot P uptake as affected by P application rates in 2011, 2012 and 2013. A, Wheat; B, Maize, C Wheat-soybean strip; D, W/M/S system. Each data point was the mean of four replicates.

Increase in P application rate increased shoot P uptake, but a changing point was observed in the relationship between P application rate and shoot P uptake (Fig 6). The plateau shoot P uptake of the W/M/S system was 71.82 kg P ha^-1^ and the critical application rate was 70 kg P ha^-1^ (Fig 6). Regarding each component of the W/M/S, the critical P application rates for wheat, maize and wheat-soybean strips were 49.7, 29.5, and 45.0 kg P ha^-1^ respectively. The plateau shoot P uptakes for wheat, maize and wheat-soybean strips were 36.82, 22.64, and 48.67 kg ha^-1^ respectively.

### Apparent recovery of fertilizer P (PRE) and apparent P balance as affected by P application rate and intercropped species

The results showed that the PRE of each crop decreased as the P application rates increased (Table 3). Compared with the 20 kg P ha^-1^ application rate, the 40, 60, and 80 kg P ha^-1^ application rates decreased PRE of wheat by 40.2%, 45.0% and 65.6% respectively. The PRE of the wheat-soybean strip was 46.5%, 39.9%, 35.7%, and 21.9% higher than that of wheat at the P application rates of 20, 40, 60, and 80 kg P ha^-1^ respectively. The PRE of maize was not significantly different among 16, 32, and 48 kg P ha^-1^ (*P*>0.05), but it was significantly higher than that of the maize applied with 64 kg P ha^-1^ (*P*<0.01). The PRE of the W/M/S system applied with more than 108 kg P ha^-1^ decreased significantly compared with that of the system applied with 36 or 72 kg P ha^-1^ (*P*<0.05), but the PRE did not differ between 36 and 72 kg P ha^-1^ (*P*>0.05) (Table 3).

**Table 3:** Apparent applied P recovery efficiency (PRE) and apparent P balance (APB) as affected by P fertilization rates and cropping systems over the three years.

Treatment	PRE (%)				APB (kg P ha^-1^)			
	Wheat	Maize	W-S	W/M/S	Wheat	Maize	W-S	W/M/S
**P**_**0**_	-	-	-	-	-28.4	-15.2	-37.2	-51.9
**P**_**1**_	33.1 a	20.7 a	48.5 a	35.9 a	-12.2	-3.7	-22.7	-26.1
**P**_**2**_	19.8 b	28.2 a	27.7 b	28.0 ab	3.5	9.3	-8.3	0.2
**P**_**3**_	18.2 b	21.5 a	24.7 b	23.2 b	23.7	25.4	11.3	36.2
**P**_**4**_	11.4 b	10.4 b	13.9 c	12.3 c	43.2	41.4	31.3	72.2
**Mean**	20.6	20.2	28.7	24.8	29.8	56.9	-25.6	30.6

Values are means of 4 replicates. Different lower-case letters indicate significant difference (*P*<0.05) by LSD between different P application rates. W-S and W/M/S indicate wheat-soybean strip and wheat/maize/soybean relay strip intercropping system respectively.

Wheat, maize and wheat-soybean strips did not achieve apparent P budget balance given each P rate (Table 3). Both P_0_ and P_1_ treatments caused P depletion in both the wheat-soybean strip and the W/M/S system, but P_2_, P_3_, and P_4_ treatment showed P surplus, although this surplus was marginal at 72 kg P ha^-1^. At higher levels of applied P, the APB increased substantially to a maximum of 56.9 kg P ha^-1^. At P_2_ rate, the W/M/S system was in apparent P balance (Table 3).

### Soil P status influenced by P application rates and cropping species

**Fig 7:**
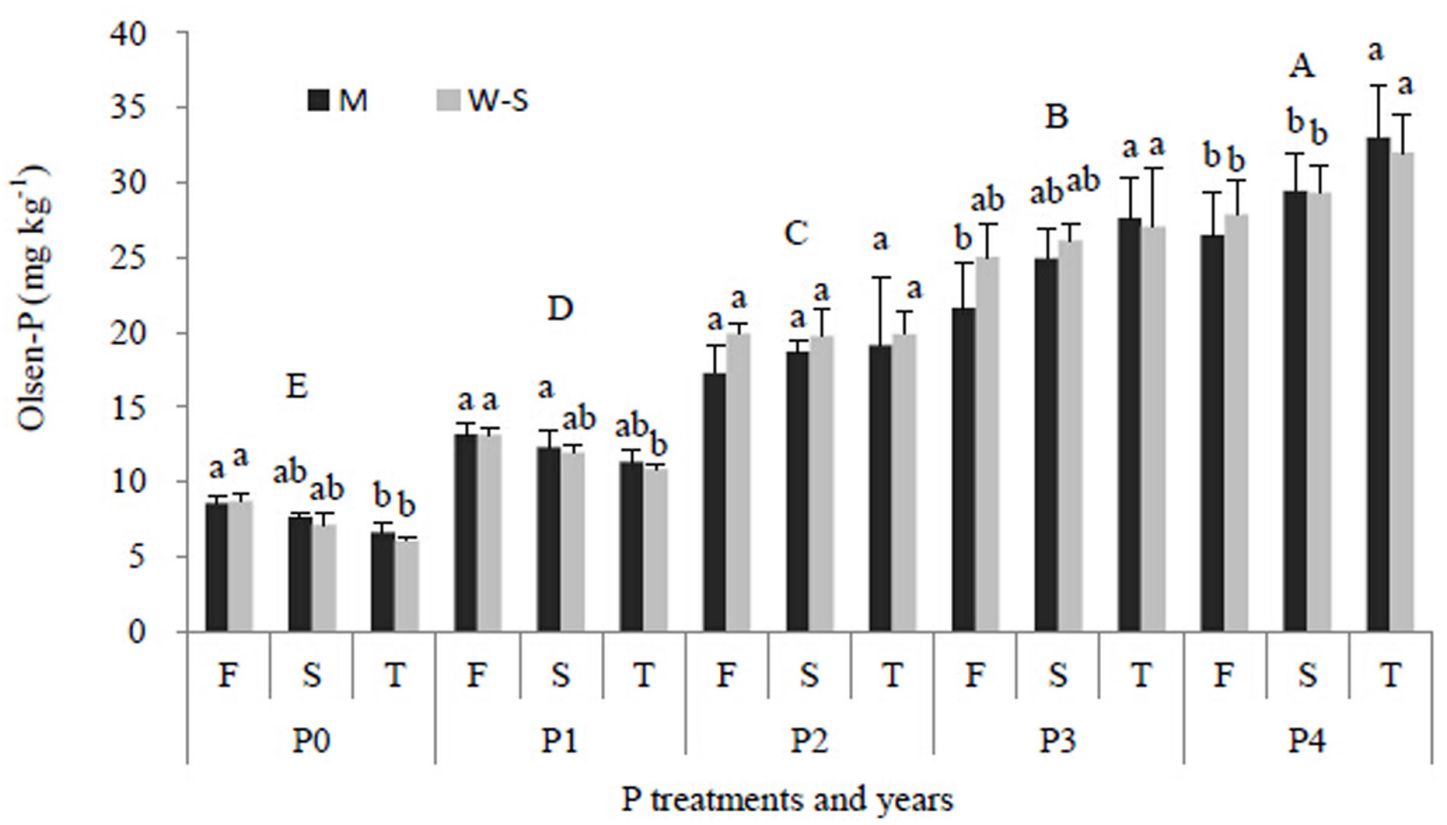
Soil Olsen-P in the top 20 cm layer of the maize strip at maize harvest (M) and the wheat-soybean strip at soybean harvest (W-S) in the W/M/S system as affected by P application rates each year. Different lower-case letters indicate significant difference (*P*<0.05) by LSD between different years under the same P application rate. Different capital letters indicate significant difference (*p*<0.05) by LSD between different P application rates. F = the first year (2011), S = the second year (2012), T = the third year (2013). P_0_, P_1_, P_2_, P_3_, P_4_ indicate the P treatments (as in table 2). Each value was the mean ± SE. Bars indicate standard errors.

Soil Olsen-P concentration was not significantly different between the wheat-soybean strip and the maize strip across all P application rates over three growing seasons (Fig 7) (*P*>0.05). Increase in P application rate increased soil Olsen-P in both the maize and wheat-soybean strips in the 0–20 cm soil layer (Fig 7). In both wheat-soybean and maize strips applied with P_2_ rate, the soil Olsen-P was consistently kept at about 19.1 mg kg^-1^ over the three years. However, both wheat-soybean and maize strips applied with P_0_ or P_1_ rates, on average, reduced Olsen-P by 2.28 and 2.02 mg kg^-1^ respectively, in 2013 compared with the Olsen-P of those strips in 2011. Contrarily, both wheat-soybean and maize strips applied with P_3_ rate increased soil Olsen-P by 2.02 and 6.03 mg kg^-1^ respectively. Wheat-soybean and maize strips applied with P_4_ rate increased soil Olsen-P by 4.17 and 6.55 mg kg^-1^ in 2013, respectively, compared with the Olsen-P of those strips in 2011 (Fig 7).

**Fig 8:**
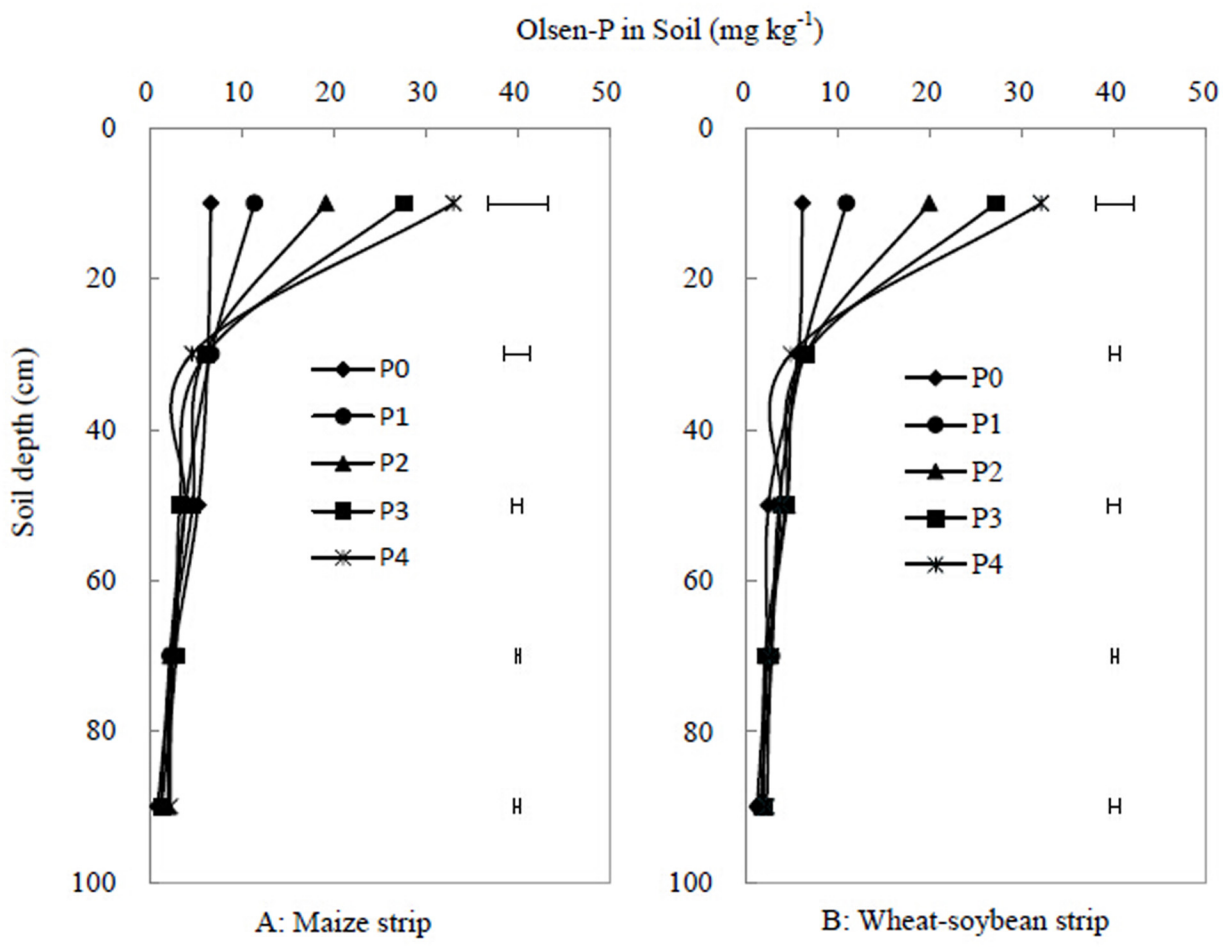
Soil Olsen-P of the maize strip at maize harvest (A) and the wheat-soybean strip at soybean harvest (B) in the 0–100 cm layers of the soil profile in 2013. The floating bars indicate LSD (*p*<0.05) between different P application rates. P_0_, P_1_, P_2_, P_3_, P_4_ indicate the P treatments (as in table 2). Each data point was the mean of four replicates.

After three years, the soil Olsen-P was greatest in the top 20 cm soil in both the maize or wheat-soybean strips, and continuous fertilizer input increased soil Olsen-P of the 0–20 cm soil but did not significantly changed across soil profile below 20 cm (Fig 8). The soil Olsen-P in 20–100 cm depths had no significant difference between maize and wheat-soybean strip across all P treatments (Fig 8).

## Discussions

### A rational P application rate enhances crop production and shoot P uptake of the W/M/S

Over-fertilization with phosphorus (P) causes severe environmental problems [24], while inadequate input of P results in low productivity and land degradation [5]. Therefore a rational P application rate is a prerequisite for a sustainable cropping system. The present study supports our hypothesis that a rational P application rate in the W/M/S system leads to a higher grain yield and more efficient P utilization.

A critical P application rate can be defined as a P application rate above which crop yield, shoot biomass and shoot P uptake do not respond to the increase in P application. Regarding the W/M/S system, the critical P application rates for grain yield, shoot biomass and P uptake were 71.5, 71 and 70 kg P ha^-1^ respectively (Figs 3–6). The W/M/S system applied with 72 kg P ha^-1^ (P_2_ treatment) had least P surplus but greatest apparent recovery of P fertilizer (PRE) (Table 3). Thus, the critical P application rate, 72 kg P ha^-1^, is sufficient for meeting the demand of producing crop grain in this system. However, the critical P application rate can be different depending on soil P concentration and yield level. Xia et al. [21] presented 40 kg P ha^-1^ as a sufficient rate for meeting P demands of all maize-based intercropping systems grown on Aridisol soil where Olsen-P is 20.3 mg kg^-1^, higher than soil P availability in this study, which is 13.2 mg/kg. However, Aulakh et al. [25] demonstrated that 60 kg P_2_O_5_ ha^-1^ or 26 kg P ha^-1^ is sufficient in a wheat/soybean system that produces 6.55 Mg ha^-1^ grain yield, much lower than the W/M/S yield in the current study (Fig 5).

A rational P application also depends on crop species and soil fertility. In the present study, the critical P application rates associated with the yield, shoot biomass and P uptake of wheat were 52.8, 51.5 and 49.7 kg P ha^-1^ respectively, and the corresponding P rates of maize were 32.0, 28.0 and 29.5 kg P ha^-1^ respectively (Figs 3–6). A study on a wheat/maize intercropping system grown on an Aridisol soil indicates a similar critical P rate, 53 kg P ha^-1^, regarding the yield and shoot P uptake of wheat [8], and 40 kg P ha^-1^ regarding those of maize [21]. However, the results are clearly different from a study in which increased P supply does not increase the average grain yield and shoot P uptake of both intercropped maize and faba bean grown on a P-deficient reclaimed desert soil [26] and also different from another work on a maize/faba bean intercropping system grown on sandy soil [27]. Thus, crops respond differently to P applications depending on various intercropping system and soil conditions [21].

### The apparent recovery efficiency of fertilizer P in W/M/S system

In China only 15–20% of the applied P is taken up by crops during the growing season [7–8], but the present results showed a greater PRE of the W/M/S system, on average 24.8%, or even higher, 28.0% when this system is applied with the critical P application rate, 72 kg P ha^-1^ (Table 3). Similarly, when the maize-based (e.g. rape, faba bean, chickpea, soybean) are supplied with their critical P rate, 40 kg P ha^-1^, the PRE is up to 30%, on average 3.8 times greater as that for the weighted means of the corresponding monocrops [21]. Interspecific interaction may be a major reason why shoot P uptake and PRE are enhanced in intercropping systems. Several studies have shown that legumes increase P acquisition of associated cereals in intercropping systems [11, 21, 26–28]. In calcareous soils, proton release by faba bean increases the P uptake of intercropped cereals [27]. The root compatibility between intercropped maize and associated faba bean may allow the intercropped maize to spread underneath the roots of neighbors and intermingle with them, thereby increasing root length density, root growth space and corresponding nutrient uptake [29]. Similarly in cereal-cereal intercropping systems, such as wheat/maize strip intercropping, roots of intercropped maize can further enlarge their growth space underneath the root zone of harvest wheat [30], thus showing clear advantage in total nutrient uptake over the sole cropping systems [11, 31]. Additionally, fertilizer placement is also important for the PRE of a cropping system, particularly for a strip relay intercropping system in which two or more crops are grown in different strips at given time. Aulakh [25] found that the PRE of soybean/wheat system was highest when 60 kg P_2_O_5_ ha^-1^ was applied to the wheat strip, followed by the treatment when the same amount of P is added into both soybean and wheat strips, and lowest when the same amount of P is supplied to the soybean strip. The results may be due to beans (e.g. soybean and faba bean) responding less to P fertilizer [26, 32] and being able to utilize the residual P left by other crops in rotation [33–34]. Similarly in the current study, the averaged PRE of the wheat-soybean strip was significantly higher than that of the pure wheat strip (Table 3). No fertilizer P was applied to soybean and the crop grown as an aftercrop could adequately utilize the residual soil P after wheat harvest. In short words, the intercropping system applied with a critical P application rate has a greater PRE than a system applied with a farmer’s common P rate, but the biological mechanisms involved are unclear and need to be examined in the future.

### Fertilizer P management strategies in W/M/S system

Most of nutrient management studies focus on nitrogen fertilizer application because nitrogen (N) is one of the most important essential nutrients [35–36] but overuse has the negative environmental effect of increasing N-related emissions from soil (e.g., N_2_O, NH_3_, NOx, and nitrate leaching) [37–39]. However, phosphorus is commonly regarded as a key driving force of water body eutrophication [40], thus reducing P efflux or keeping P apparent balance is crucial in reducing water pollution, especially at the hilly area [41], such as the Southwest of China. Better P fertilizer management for cropping systems can maintain P balance of inputs and output [6]. In the present W/M/S system, the critical P application rate (72 kg P ha^-1^) is lower than the conventionally recommended P application rate (85.2 kg P ha^-1^) [42], but it meets the nutrient requirement of crop producing high grain yield (Figs 4 and 5). This yield is even higher than the yield achieved by the conventionally recommended P application rate [43]. Thus, the critical P application rate results in efficient nutrient uptake while less nutrient output and consequently maintain P apparent balance. After three annual growing seasons, the critical P application rate of 72 kg P ha^-1^ maintained the soil Olsen-P concentration, 19.1 mg kg^-1^ (Fig 7), with lower P input causing soil P depletion, and higher P input resulting in excessive P accumulation in soil. Thus, the dominant cropping system maintains P input/output balance and the soil available P at an appropriate level, and the P efflux from the arable field could consequently be mitigated. This is vital for protecting the ecological environment and developing sustainable crop production intensification in the Southwest of China, the upstream of the Yangtze River.

## Conclusions

Utilization of the different P requirement by the three crops and a rational P management strategy may have contributed to enhanced total grain yields and P use efficiency of the W/M/S system. Consequently, the overall apparent recovery of fertilizer P (PRE) of the W/M/S system is greatly increased comparing with the national average. A rational P application rate (e.g. wheat 40 kg P ha^-1^/maize 32 kg P ha^-1^/soybean 0 kg P ha^-1^) in the W/M/S system resulted in a balanced P input and output, high crop yields and high P recovery efficiency (28.8%) while maintaining a steady soil Olsen-P level (19.1 mg kg^-1^). The W/M/S system with a rational P application rate provides the basis for a sustainable and productive agricultural system.

## Supporting Information

S1 Fig Wheat/maize/soybean relay strip intercropping system. The pattern of the W/M/S system is shown as pictures (A, B, C, D, E). Wheat is first sown in November but maize strip is blank (A). Maize was transplanted in April of the following year when wheat is at flowering stage (B). After wheat is harvested in May of the following year soybean is planted on wheat strip after wheat harvest (C, D). Thus, soybean is planted as relay crop following wheat (E).
(RAR)

## References

[1] FAO (2011) FAOSTAT Database-Agriculture Production. Food and Agriculture Organization of the United Nations, Rome.

[2] Zhang FS, Cui ZL, Chen XP, Ju XT, Shen JB, Chen Q, et al (2012) Integrated nutrient management for food security and environmental quality in China. Adv Agron 116:1–40.

[3] Ma L, Ma WQ, Velthof GL, Wang FH, Qin W, Zhang FS, et al (2010) Modeling nutrient flows in the food chain of China. J Environ Qual 39:1279–1289. 20830916 10.2134/jeq2009.0403

[4] Ma WQ, Ma L, Li JH, Wang FH, Sisak I, Zhang FS (2011) Phosphorus flows and use efficiencies in production and consumption of wheat, rice, and maize in China. Chemosphere 84: 814–821. doi: 10.1016/j.chemosphere.2011.04.055 21570104 10.1016/j.chemosphere.2011.04.055

[5] Vitousek PM, Naylor R, Crews T, David MB, Drinkwater LE, Holland E, et al (2009) Nutrient imbalances in agricultural development. Science 324: 1519–1520 doi: 10.1126/science.1170261 19541981 10.1126/science.1170261

[6] Li H, Huang G, Meng L, Ma L, Yuan L, Wang F, et al (2011a) Integrated soil and plant phosphorus management for crop and environment in China. A review. Plant Soil 349:157–167.

[7] Zhang L, vander WW, Bastiaans L, Zhang S, Li B, Spiert JHJ (2008a) Light interception and utilization in relay intercrops of wheat and cotton. Field Crop Res 107: 29–42.

[8] Zhang WF, Ma WQ, Ji YX, Fan MS, Oenema O, Zhang FS (2008b) Efficiency, economics, and environmental implications of phosphorus resource use and the fertilizer industry in China. Nutr Cycl Agroecosyst 80: 131–144.

[9] Zhong X, Zhao X, Bao H, Li HH, Li GT, Lin QM (2004) The evaluation of phosphorus leaching risk of 23 Chinese soil, I. Leaching criterion. Acta Ecol Sin 24: 2275–2280.

[10] Chinese Ministry of Environment Protection, 2010. Available: http://www.gov.cn/jrzg/2010-02/10/content_1532174.htm.

[11] Li L, Sun JH, Zhang FS, Li XL, Yang SC, Rengel Z (2001a) Wheat/maize or soybean strip intercropping. I. Yield advantage and interspecific interactions on nutrients. Field Crop Res 71:123–137.

[12] Li L, Sun JH, Zhang FS, Li XL, Rengel Z, Yang SC (2001b) Wheat/maize or soybean strip intercropping. II. Recovery or compensation of maize and soybean after wheat harvesting. Field Crop Res 71:173–181.

[13] Mao L, Zhang L, Li W, Werf WV, Sun J, Spiertz H, et al (2012) Yield advantage and water saving in maize/pea intercrop. Field Crop Res 138: 11–20.

[14] Zhang XB, He XB, Wen AB, Walling DE, Feng MY, Zou X (2004) Sediment source identification by using ^137^Cs and ^210^Pb radionuclides in a small catchment of the Hilly Sichuan Basin, China. Chinese Sci Bull 49: 1953–1957.

[15] Yang F, Huang S, Gao RC, Liu WG, Yong TW, Wang XC et al (2014) Growth of soybean seeding in relay strip intercropping systems in relation to light quantity and red: far-red ratio. Field Crop Res 155: 245–253.

[16] Chen YX, Chen XH, Tang YQ, Zhang FS, Chen XP, Zhang CC, et al (2014a) Effect of nitrogen on dry matter accumulation and yield in wheat/maize/soybean intercropping systems. Acta Prataculturae Sinica 23: 73–83.

[17] Chen XH, Xu KW, Tang YQ, Liu J, Chen XP, Zhang CC, et al (2014b) Nitrogen accumulation, allocation and translocation in wheat/maize/soybean relay intercropping system. J Plant Nutr Fertil 20: 1127–1138.

[18] Blake GR, Hartge KH (1986) Bulk density In: Klute A. (ed.), Methods of Soil Analysis.Part 1. Physical and Mineralogical Methods. Agronomy Monograph, Soil Sci Soc Am (SSSA), Madison, WI vol. 9,2nd ed., pp. 363–375.

[19] Page AL (1982) Methods of soil analysis (Part 2), 2nd edn American Society of Agronomy, Madison.

[20] Murphy J, Riley J (1962) A modified single solution method for the determination of PO% innatural waters. Anal Chim Acta 27:31–36.

[21] Xia HY, Wang ZG, Zhao JH, Sun JH, Bao XG, Christie P, et al (2013) Contribution of interspecific interactions and phosphorus application to sustainable and productive intercropping systems. Field Crop Res 154: 53–64.

[22] Zhang H, Xu M, Shi X, Li Z, Huang Q, Wang X (2010) Rice yield, potassium uptake and apparent balance under long-term fertilization in rice-based cropping systems in southern China. Nutr Cycl Agroecosyst 88: 341–349.

[23] Tang X, Ma YB, Hao XY, Li XY, Li JM, Huang SM, et al (2009) Determining critical values of soil Olsen-P for maize and winter wheat from long-term experiments in China. Plant Soil 323: 143–151.

[24] Le C, Zha Y, Li Y, Sun D, Lu H, Yin B (2010) Eutrophication of lake waters in China: Cost, causes, and control. J Environ Manage 45: 662–668. 10.1007/s00267-010-9440-3 20177679

[25] Aulakh MS, Pasricha NS, Bahl DS (2003) Phosphorus fertilizer respond in an irrigated soybean-wheat production system on a subtropical, semiarid soil. Field Crop Res 80: 99–109.

[26] Mei PP, Gui LG, Wang P, Huang JC, Long HY, Christie P, et al (2012) Maize/faba bean intercropping with rhizobia inoculation enhances productivity and recovery of P in a reclaimed desert soil. Field crop Res 130: 19–27.

[27] Li L, Li SM, Sun JH, Zhou LL, Bao XG, Zhang HG, et al (2007) Diversity enhances agricultural productivity via rhizosphere phosphorus facilitation on phosphorus-deficient soils. P Natl Acad Sci USA 104:11192–11196. 10.1073/pnas.0704591104 PMC1899187 17592130

[28] Li HG, Shen JB, Zhang FS, Marschner P, Cawthray G, Rengel Z (2010) Phosphorus uptake and rhizosphere properties of intercropped and monocropped maize, faba bean, and white lupin in acidic soil. Biol Fertil Soils 46:79–91.

[29] Li L, Sun JB, Zhang FS, Guo TW, Bao XG, Andrew FA, et al (2006) Root distribution and interactions between intercropped species. Oecologia 147: 280–290. 16211394 10.1007/s00442-005-0256-4

[30] Li L, Sun JH, Zhang FS (2011b) Intercropping with wheat leads to greater root weight density and larger below-ground space of irrigated maize at late growth stages. Soil Sci Plant Nutr 57: 61–67.

[31] Li QZ, Sun JH, Wei XJ, Christie P, Zhang FS, Li L (2011c) Overyielding and interspecific interactions mediated by nitrogen fertilization in strip intercropping of maize with faba bean, wheat and barley. Plant Soil 339: 147–161.

[32] Vandamme E, Pypers P, Vanlauwe B, Baijukya F, Smolders E, Merckx R (2014) Residual phosphorus effects and nitrogen × phosphorus interactions in soybean-maize rotations on a P-deficient Ferralsol. Nutr Cycl Agroecosyst 98: 187–201.

[33] Zingore S, Murwira HK, Delve RJ, Giller KE (2008) Variable grain legume yields, responses to phosphorus and rotational effects on maize across soil fertility gradients on African smallholder farms. Nutr Cycl Agroecosyst 80:1–18.

[34] Kihara J, Vanlauwe B, Waswa B, Kimetu JM, Chianu J, Bationo A (2010) Strategic phosphorus application in legume-cereal rotations increases land productivity and profitability in Western Kenya. Exp Agric 46:35–52.

[35] Silvia RM, Tommaso M (2013) Evaluation of nitrogen management in maize cultivation grows on soil amended with sewage sludge and urea. Eur J Agron 45: 59–67.

[36] Xu JZ, Liao LX, Tan JY, Shao XH (2013) Ammonia volatilization in gemmiparous and early seedling stages from direct seeding rice fields with different nitrogen management strategies: A pots experiment. Soil Till Res 126: 169–176.

[37] Erisman JW, Bleeker A, Hensen A, Vermeulen A (2008) Agricultural air quality in Europe and the future perspectives. Atmos Environ 42: 3209–3219.

[38] Robertson JW and Vitousek PM (2009) Nitrogen in Agriculture: Balancing the Cost of an Essential Resource. Annu Rev Environ Resourc 34: 97–125.

[39] Cui ZL, Yue SC, Wang GL, Mang QF, Wu L, Yang ZP, et al (2013) Closing the yield gap could reduce projected greenhouse gas emissions: a case study of maize production in China. Global Change Biol 19: 2467–2477 10.1111/gcb.12213 23553871

[40] Schindler D W, Hecky RE, Findlay DL, Stainton MP, Parker BR, Paterson MJ, et al (2008) Eutrophication of lakes cannot be controlled by reducing nitrogen input: results of a 37-year whole-ecosystem experiment. P Natl Acad Sci USA 105: 11254–11258. 10.1073/pnas.0805108105 PMC2491484 18667696

[41] Gao Y, Zhu B, Zhou P, Tang JL, Wang T, Miao CY (2009) Effects of vegetation cover on phosphorus loss from a hill slope cropland of purple soil under simulated rainfall: a case study in China. Nutr Cycl Agroecosys 85: 263–273.

[42] DB51/T810-2008 The relay-planting system of “wheat/corn/soybean”. Available: http://www.rcsoybean.com/.

[43] Yong TW (2009) Analysis of the nitrogen uptake and utilization, rhizosphere micro-ecology in the “wheat/maize/soybean” relay-cropping system. Dissertation, Sichuan Agricultural University. Available: http://acad.cnki.net/Kns55/brief/result.aspx?dbPrefix=CDFD.

